# Efficacy and safety clinical trial with efavirenz in patients diagnosed with adult Niemann-pick type C with cognitive impairment

**DOI:** 10.1097/MD.0000000000031471

**Published:** 2022-12-02

**Authors:** Jordi Gascón-Bayarri, Petru Cristian Simon, Roser Llop, Thiago Carnaval, María Dolores Ledesma, Imma Rico, Cristina Sánchez-Castañeda, Jaume Campdelacreu-Fumadó, Nahum Calvo-Malvar, Mònica Cos, Eugenia de Lama, Montserrat Cortés-Romera, Laura Rodríguez-Bel, Celia Pérez-Sousa, María Cerdán Sánchez, Nuria Muelas, María Dolores Sevillano, Pablo Mir, Adolfo López de Munain, Anna Ferrer, Sebastián Videla

**Affiliations:** a Neurology Department, Bellvitge University Hospital, L’Hospitalet DE Llobregat, Barcelona, Spain; b Adult Lysosomal Diseases Clinical Expertise Unit, Bellvitge University Hospital, L’Hospitalet DE Llobregat, Barcelona, Spain; c Bellvitge Biomedical Research Institute (IDIBELL), L’Hospitalet DE Llobregat, Barcelona, Spain; d Department of Morphological Sciences, School of Medicine, Autonomous University of Barcelona, Bellaterra, Spain; e Clinical Pharmacology Department, Bellvitge University Hospital, L’Hospitalet de Llobregat, Barcelona, Spain; f Pharmacology Unit, Department of Pathology and Experimental Therapeutics, School of Medicine and Health Sciences, IDIBELL, University of Barcelona, L’Hospitalet DE Llobregat, Barcelona, Spain; g Severo Ochoa Molecular Biology Center (CSIC-UAM), Madrid, Spain; h Department of Clinical Psychology and Psychobiology, Neurosciences Institute, University of Barcelona, Barcelona, Spain; i Neuroradiology Department, Bellvitge University Hospital, L’Hospitalet DE Llobregat, Spain; j Nuclear Medicine-PET/CT Department (IDI). Bellvitge University Hospital, L’Hospitalet DE Llobregat, Spain; k Neurology Department, A Coruña University Hospital Complex, A Coruña, Spain; l Neurology Department, Santa Lucía-Santa María Rosell University Hospital, Cartagena, Spain; m Neurology Department, La Fe University and Polytechnical Hospital, Valencia, Spain; n Neurology Department, Salamanca University Hospital, Salamanca, Spain; o Movement Disorders Unit, Neurology and Clinical Neurophysiology Department, Seville Biomedical Institute, Virgen del Rocío University Hospital/CSIC/University of Seville, Seville, Spain; p Neurodegenerative Diseases Network of Biomedical Research Centers (CIBERNED), Madrid, Spain; q Medicine Departament, School of Medicine, University of Seville, Seville, Spain; r Neurology Department, Donostia-Osakidetza University Hospital, San Sebastian, Spain; s Neuroscience Department, Biodonostia Institute, San Sebastian, Spain; t Neuroscience Department, University of the Basque Country, San Sebastian, Spain; u Pharmacy Department, Bellvitge University Hospital, L’Hospitalet de Llobregat, Barcelona, Spain; v Clinical Research Support Unit (HUB-IDIBELL: Bellvitge University Hospital & Bellvitge Biomedical Research Institute), Clinical Pharmacology Department, Bellvitge University Hospital, L’Hospitalet de Llobregat, Barcelona, Spain.

**Keywords:** cognitive impairment, efavirenz, Niemann-pick disease type C, NPC1

## Abstract

Niemann-Pick disease Type C (NPC) is a genetic, incurable, neurodegenerative disorder. This orphan disease is most frequently caused by mutations in the NPC1 protein, resulting in intralysossomal cholesterol accumulation. NPC1 is found in neuronal cell bodies, axon terminals and synaptosomes, suggesting it plays a role in lysosomal degradation pathway and in synaptic transmission. Neuronal function is especially vulnerable to NPC1 deficiency and synaptic changes seem a key element in disease development. Currently, Miglustat (Zavesca®) is the only approved treatment for NPC. However, preclinical evidence showed that low-dose Efavirenz reverted synaptic defects through pharmacological activation of the enzyme CYP46.

**Methods:**

This is a single-center, phase II clinical trial to evaluate the efficacy and safety of Efavirenz in addition to standard of care in patients diagnosed with adult or late juvenile-onset NPC with cognitive impairment. All enrolled patients will be treated orally with 25 mg/d of Efavirenz for 52 weeks (1 year). Secondary objectives include evaluating clinical (neurological and neuropsychological questionnaires) and biological (imaging and biochemical biomarkers) parameters.

**Discussion:**

NPC is still an unmet medical need. Although different therapeutic approaches are under study, this is the first clinical trial (to the best of our knowledge) studying the effects of Efavirenz in adult- and late-juvenile-onset NPC. Despite the small sample size and the single-arm design, we expect the results to show Efavirenz’s capacity of activating the CYP46 enzyme to compensate for NPC1 deficiency and correct synaptic changes, therefore compensating cognitive and psychiatric changes in these patients. This study may provide direct benefit to enrolled patients in terms of slowing down the disease progression.

## 1. Introduction

### 1.1. Background and rationale

Niemann-Pick disease Type C (NPC) is a genetic, neurodegenerative disorder with a life expectancy ranging between 1 – and 30 years after diagnosis. From a clinical perspective, NPC usually affects the viscera (splenomegaly and/or hepatomegaly), but it is, above all, a neurological disease. It is a progressive disease without curative treatment and with only 1 treatment approved to date – with limited efficacy (Miglustat, Zavesca®). In 2019, adult-onset NPC prevalence in Spain was 27 patients, of which ten are being treated at Bellvitge University Hospital (BUH).

The disease is classified according to the age of onset of the neurological symptoms in: early infantile (before 2 years of age); late infantile (between 3 and 5 years of age); early juvenile (between 5 and 14 years of age); late juvenile (between 14 and 16 years of age); and adult (from 16 years of age onwards).

The clinical presentation includes psychomotor development regression, epilepsy, ataxia, vertical supranuclear ophthalmoplegia, bulbar syndrome, dystonia, splenomegaly, cognitive impairment, behavioral disorders, and symptoms of psychiatric disorders, with behavioral/cognitive symptoms being most common in adults. Altogether, these symptoms point towards synaptic changes as a key element in disease development, similar to other dementias (e.g., Alzheimer’s), where cognitive impairment correlates with the loss of synapses.

In 95% of the cases, genetic mutations in the NPC1 protein are responsible for disease development. The NPC1 protein is involved in the intracellular transport of cholesterol.^[1–3]^ From a cellular and molecular perspective, one of the main hallmarks of NPC is the accumulation of cholesterol and sphingolipids in lysosomal compartments, where NPC1 is found.^[4]^ Although lipid accumulation occurs in all cell types, neurons are especially vulnerable to NPC1 deficiency, suggesting that neuronal function is especially related to this protein. Also, NPC1 is found not only in cell bodies, where endosomes and lysosomes are highly abundant,^[5]^ but also in axon terminals and synaptosomes,^[6,7]^ pointing towards a synaptic role from NPC1 other than participating in the lysosomal degradation pathway.

Some authors demonstrated the importance of cholesterol synaptic redistribution and elimination for the correct development of long-term potentiation (LTP).^[8]^ LTP is a major event for synaptic plasticity, involved in processes like memory, learning, and emotional responses. Cholesterol loss during LTP has been associated with the enzyme CYP46A1, mobilized to the plasmatic membrane in response to synaptic stimulation, hydroxylating cholesterol, making it more soluble, and mediating the main pathway for its degradation in the brain.^[9,10]^

Given that NPC1 can bind cholesterol and participate in intracellular cargo mobilization, we hypothesized that NPC1 would have a synapse-specific role in redistributing cholesterol and mobilizing the CYP46A1 enzyme to the synaptic membrane, required for LTP. We believe that the pharmacological activation of CYP46A1 could compensate for NPC1 deficiency and correct synaptic changes, therefore compensating cognitive and psychiatric alterations in patients with NPC.

Preclinical data on Npc^nmf164^ mice^[11]^ (which have the D1005G mutation in the NPC1 gene and develop NPC slower than NPC1^−/−^ mice) have provided evidence that low-dose Efavirenz (EFV) (compared to the dosages used with antiretrovirals) modifies the morphological, molecular, and functional changes in their synapses.^[12]^ It also improved cognitive and psychiatric changes, reverted synaptic defects through pharmacological activation of the enzyme CYP46, and increased the life expectancy of Npc^nmf164^ mice by 30%.^[12]^ These encouraging results are the rationale for conducting this clinical trial on this orphan disease.

According to the summary of product characteristics (SmPC), EFV was authorized in May 1999 as a non-nucleoside reverse transcriptase antiretroviral inhibitor and, currently, it is a generic drug used in the combined antiviral treatment of human immunodeficiency virus-1^[13]^ in infected adults, adolescents and children aged 3 months and older that weigh at least 3.5 kg. In adults, the recommended oral dose of EFV combined with nucleoside reverse transcriptase inhibitors with or without a protease inhibitor is 600 mg once a day (600 mg/d). It is a treatment administered “for life” in patients infected with HIV.

This clinical trial aims to evaluate the efficacy and safety of EFV at an oral dose of 25 mg/d in patients diagnosed with adult or late juvenile-onset NPC with cognitive impairment.

### 1.2. Explanation for the choice of comparators

Given the clinical trial characteristics, all patients included in the clinical trial will be treated orally with 25 mg/d of EFV for 52 weeks (1 year), in addition to Standard of Care (SoC).

### 1.3. Objectives

The primary, secondary, and safety objectives are summarized in Table 1.

**Table 1:** Summarized study objectives.

Primary Objective	
•To evaluate the efficacy* and safety of EFV at an oral dose of 25 mg/d, in addition to standard treatment, in patients diagnosed with adult or late juvenile-onset NPC with cognitive impairment.	
*Note: Given that adult or late juvenile-onset NPC is rare and causes progressive cognitive impairment, treatment with EFV will be considered effective if it “slows down or stops cognitive impairment in patients diagnosed with adult or late juvenile-onset NPC with cognitive impairment.” Meaning, it is hoped that after 1 year of treatment, these patients remain at the baseline with respect to cognitive impairment.	
Secondary objectives	
**Clinical**	• Neurological: To evaluate the change in the following questionnaires/scales:
	SARA
	EAT-10 score.
	PDS
	• Neuropsychological (protocol used in clinical practice): To evaluate the change in the following questionnaires/scales:
	CDR-SoB
	Verbal memory: FCSRT
	a-BT
	Verbal fluency: Semantic and phonetic cues
	TMT A and B
	STROOP
	WAIS and the block design, symbol search and vocabulary subtests (WAIS-III/WAIS-IV)
	BNT, and the Naming and Semantic Knowledge subtests
	JLO
	DEX
	NPI
	AES
	BDI
	C-SSRS
**Biological**	• To evaluate the change in functional and imaging biomarkers related to NPC:
	^18^FDG PET—scan
	Brain MRI
	Abdominal ultrasound
	Oculography
	• Evaluate the change in biochemical biomarkers related to NPC:
	Oxysterols
	Lyso-SM-509
	24-OH-Cholesterol
	Beta-Amyloid, Tau, and phosphorylated Tau
**Safety**	• To evaluate the safety of EFV in patients diagnosed with adult or late juvenile-onset NPC with cognitive impairment.

18 FDG PET—scan = ^18^Fluorodeoxyglucose Positron emission tomography scan, a-BT = abbreviated Barcelona test, AES = apathy evaluation scale, BDI = Beck’s depression inventory, BNT = Boston naming test, CDR-SoB = clinical dementia rating scale-sum of boxes, C-SSRS = Columbia suicide severity rating scale, DEX = dysexecutive questionnaire, EAT-10 = the eating assessment tool score, EFV = efavirenz, FCSRT = free and cued selective reminding test, JLO = judgement of line orientation test, MRI = magnetic resonance imaging, NPC = Niemann-Pick disease type C, NPI = neuropsychiatric inventory, PDS = Pineda disability scale, SARA = scale for the assessment and rating of ataxia, STROOP = stroop color and word test, TMT = trail making test, WAIS = Wechsler adult intelligence scale.

### 1.4. Trial design

This is a single-center, phase II clinical trial to evaluate the efficacy and safety of EFV, in addition to SoC, in patients diagnosed with adult or late juvenile-onset NPC with cognitive impairment.

All enrolled patients will be treated orally with 25 mg/d of EFV for 52 weeks (1 year), in addition to SoC. According to the SmPC, the recommended dose of EFV as an antiretroviral for adults is 600 mg/d orally.

## 2. Methods: Participants, interventions, and outcomes

### 2.1. Study setting

This is a single-center clinical trial. Therefore, this study will be carried out at BUH (L’Hospitalet de Llobregat, Barcelona, Spain) and will be coordinated by the Neurology Department. This is a fully equipped tertiary hospital.

### 2.2. Eligibility criteria

Patients that are to be enrolled in the trial must meet all the inclusion criteria and none of the exclusion criteria.

#### 2.2.1. Inclusion criteria.

Patients must:

• Be at least 14 years of age, of both genders.
• Be diagnosed with adult-onset NPC based on a genetic study with 2 genetic mutations related to this disorder OR 1 genetic change, 1 positive filipin stain, and a positive biomarker.
• Have mild to moderate cognitive impairment with a global Clinical Dementia Rating (CDR) score of 0.5 to 2 and/or a CDR-SoB ≤ 12.
• Have the capacity (at the investigator’s discretion) to understand the nature and risks of the trial and the relevant information given, the capacity to decide whether to participate, and sign the written informed consent.
• Have a live-in caregiver available more than 5 days a week that can inform the patient’s medical situation, understand the nature and risks of the trial, and sign the written informed consent.
• Be proficient in Spanish (the language in which cognitive tests will be conducted).
• Have sufficient visual and auditory acuity for understanding explanations given and neuropsychological tests.
• Have a minimum 8 years of schooling.
• Be on Miglustat treatment (only treatment indicated for NPC) with a stable dose for the last 3 months.
• In the case of women of child-bearing potential (i.e., the period between menarche and menopause), have a negative pregnancy test.
• In the case of women of child-bearing potential, commit to use highly effective contraceptive measures, which include barrier methods in combination with other contraceptive measures (e.g., oral, or other hormonal contraceptives) or commit to sexual abstinence until at least 12 weeks after the final dose of EFV.
• In the case of male patients, commit to use barrier contraceptives (condoms) until at least 12 weeks after the final dose of EFV.
• Sign the written informed consent.

***Note***: Although the primary target population of the study is patients with adult-onset NPC (i.e., patients > 16 years old), we will also enroll patients aged between 14 and 16 years old (patients with late juvenile-onset NPC). The justification for including the late juvenile population (between 14 and 16 years old) in the study is that NPC also causes cognitive impairment in this age interval.

#### 2.2.2. Exclusion criteria.

• Any sign or suspicion of a diagnosis other than NPC.
• Active Central Nervous System infections that may interfere with normal brain function.
• History of severe traumatic head injury.
• History of severe psychiatric illnesses: bipolar disorder, schizophrenia, major depressive disorder.
• Risk of suicide, according to the live-in caregiver or investigator.
• History of substance abuse or dependence.
• Presence of cerebrovascular disease that can explain the cognitive impairment (in the investigator’s opinion).
• Inability to tolerate the study-related procedures.
• Stage IV chronic kidney disease.
• Chronic liver disease or increased Aspartate Aminotransferase or Alanine Aminotransferase levels (≥3 times the upper limits of normal).
• Chronic viral hepatitis B or C.
• Patients with systemic immunosuppression.
• Unstable epilepsy (at the investigator’s discretion) in the last 3 months*.

*Note: Those with controlled/stable epilepsy can be enrolled.

• History of malignancies not considered stable or cured (in general, patients who have not been treatment-free for at least 5 years before screening and considered to be at low risk of recurrence by the treating physician).
• Severe dementia that hinders the completion of cognitive tests and evaluations.
• History of allergy/hypersensitivity to EFV or to any of the excipients found in the formula.
• Patients using anticoagulants are not excluded from the trial but will NOT be offered the possibility to join the lumbar puncture group.
• Concomitant treatment with any of the following: terfenadine, astemizole, cisapride, midazolam, triazolam, pimozide, bepridil, or ergot alkaloids (e.g., ergotamine, dihydroergotamine, ergonovine, and methylergonovine)*.

*Note: These drugs are substrates of the CYP3A4 that could cause a competitive inhibition of the EFV metabolism and generate serious and/or life-threatening AEs (e.g., cardiac arrhythmias, prolonged sedation, respiratory depression).

• Co-administration with elbasvir and grazoprevir due to the potential of EFV to significantly decrease plasma concentrations of both drugs.
• Use of herbal medicinal products containing St. John’s Wort (*Hypericum perforatum*) due to the risk of decreasing the plasma concentrations and the clinical effects of EFV.
• Family history of sudden death or congenital prolongation of the QTc interval or any other clinical condition known to prolong the QTc interval.
• Family history of symptomatic cardiac arrhythmias, clinically relevant bradycardia, or congestive heart failure associated with a reduced left ventricular ejection fraction.
• Severe disturbances of electrolyte balance, such as hypokalemia or hypomagnesemia.
• Concomitant treatment with drugs known to prolong the QTc interval, such as class IA and class III antiarrhythmics; neuroleptics; antidepressant drugs; certain antibiotics (i.e., macrolides, fluoroquinolones); imidazole and triazole antifungal agents; certain non-sedating antihistamines (i.e., terfenadine, astemizole); cisapride; flecainide; certain antimalarials; and methadone.

### 2.3. Interventions

The study intervention is EFV. All patients will be treated orally with 25 mg/d of EFV for 52 weeks (1 year), in addition to SoC. Despite this established 25 mg/d dose, successive blood analyses will be performed during the trial, and those patients with no evidence of a blood 24-OH-Cholesterol increase ≥ 1.5 times their baseline levels will have their EFV dose increased up to 200 mg/d.

According to the SmPC, the recommended antiretroviral dose of EFV for adults is 600 mg/d PO, combined with nucleoside reverse transcriptase antiretroviral inhibitor with or without a protease inhibitor. The dose selected for this trial (25 mg/d) is the 1 administered to the pediatric population with 3.5 to <5 kg of weight. Also, as recommended by the SmPC, patients will be advised to take the medication at bedtime.

#### 2.3.1. Rationale for dose selection.

Preclinical studies using NPC mouse models used an Efavirenz dose 300 times smaller than the 1 used in HIV patients.^[14]^ Although very small, the dose used has been proven to activate CYP46 and efficiently promoted the recycling of cholesterol in the brains of normal mice.^[14]^

However, extrapolating preclinical doses to a clinical setting is challenging. The significant differences in body size, brain size, and the blood-brain barrier must be considered, among other things. Also, administering humans with a dose as low as the 1 administered in mice would risk not having the desired effect.

Notably, 1 clinical trial (NCT03706885, ClinicalTrials.gov) is currently being conducted in patients with Alzheimer’s dementia using oral doses of EFV of 50 and 200 mg/d to activate CYP46 in the brain.

In light of all that, the study team has selected a dose of 25 mg/d of EFV, considering that the desired effect should be observed with this dose – a dose 24 times smaller than the recommended antiretroviral dose for HIV patients (600 mg/d). Given that increases in plasma 24-OH-Cholesterol concentrations (≥1.5 times the pre-EFV baseline values) have been observed in the preclinical studies, we will perform successive blood tests (every 3 months). If no 24-OH-Cholesterol increases ≥ 1.5 times the pre-EFV baseline values are observed, we shall consider increasing the dose to up to 200 mg/d.

### 2.4. Criteria for discontinuing or modifying allocated interventions

As mentioned before, all enrolled patients will receive EFV + SoC. The EFV will be discontinued if the investigator deems that it poses an unacceptable risk to the patient; the patient withdraws his consent; there is a significant violation of the study protocol; there is a loss to follow-up; or in case a study participant becomes pregnant.

### 2.5. Strategies to improve adherence to intervention

In addition to collecting and reviewing empty medication boxes, patients will be provided with a “Patient’s diary” for better controlling treatment adherence.

### 2.6. Relevant concomitant care permitted or prohibited during the trial

As mentioned in the exclusion criteria, the following concomitant medications are prohibited during the trial:

• CYP3A4 inhibitors, including, but not limited to terfenadine, astemizole, cisapride, midazolam, triazolam, pimozide, bepridil, or ergot alkaloids (e.g., ergotamine, dihydroergotamine, ergonovine, and methylergonovine). Substrates that compete with EFV for CYP3A4-binding could decrease its metabolism and induce serious and/or life-threatening adverse reactions (e.g., arrhythmias, prolonged sedation, or respiratory depression).
• Elbasvir and Grazoprevir, because EFV has the potential to significantly decrease plasma concentrations of both drugs.
• Herbal medicinal products containing St. John’s Wort (*Hypericum perforatum*) due to the risk of decreasing plasma concentrations and the clinical effects of EFV.
• Drugs known to prolong the QTc interval, including, but not limited to class IA and class III antiarrhythmics; neuroleptics; antidepressant agents; certain antibiotics (e.g., macrolides, fluoroquinolones, and imidazole and triazole antifungal agents); certain non-sedating antihistamines (terfenadine, astemizole); cisapride; flecainide; certain antimalarials; and methadone.

### 2.7. Outcomes:

Primary and secondary outcomes are summarized in Table 2.

**Table 2:** Summarized outcome measures.

Primary outcome measure	
Change (not deterioration) in cognitive performance at 52 wks of starting treatment with EFV, assessed by:	
CDR-SoB score.	
FCSRT score (to evaluate verbal memory).	
The executive functions as per the following:	
a)a-BT scores in the digit span subtest.	
b)a-BT scores in the mental control subtest.	
c)Scores in verbal fluency subtest: semantic and phonemic tasks.	
d)TMT A and B scores	
e)SCWT scores.	
Secondary outcome measures	
**Clinical**	Neurological: Changes at 52 wks of starting treatment with EFV relative to baseline in:
	SARA score
	EAT-10 score.
	PDS score
	• Neuropsychological (protocol used in clinical practice): In general, the scaled score will be determined for the following questionnaires/scales:
	CDR-SoB^*^ score.
	*Note: It offers a specific score from 0 to 18, the higher the score the greater the severity.
	FCSRT score
	a-BT assessment in the following domains:
	◦Orientation, digit span, automatized sequences (e.g., counting to 20, d of the wk, mo of the yr), comprehension of verbal commands, and comprehension of complex verbal material (i.e., yes-or-no answers to BDAE-based questions) subtests’ scores.
	◦Bilateral gesture-based limb praxis test
	◦Visual memory (i.e., figures)
	◦Reading, repeating, and writing
	◦Mental and written arithmetic
	◦Reciprocal coordination
	◦Unilateral alternating sequences subtest (e.g., serial hand tests, such as fist-palm-side, tapping).
	◦Constructional praxis (drawing copy).
	Verbal Fluency score: Semantic and phonemic tasks
	TMT A and B scores
	SCWT score
	WAIS-III/WAIS IV
	BNT, Naming and Semantic Knowledge subtests’ scores
	JLO Test score.
	DEX questionnaire score.
	NPI score.
	AES score.
	BDI score.
	C-SSRS score.
**Biological**	• Brain ^18^FDG PET–Scan: Quantification of cerebellar, cortical, and thalamic metabolism.
	• Brain MRI: Cortical, white matter, basal ganglia, and cerebellar structures volumetric analysis.
	• Abdominal ultrasound: Spleen size (cm).
	• Oculography: initial speed, amplitude, and execution speed of vertical and horizontal saccades
	• Plasma Oxysterols levels
	• Plasma Lyso-SM-509 level
	• Plasma 24-OH-Cholesterol level
	• Cerebrospinal Fluid (CSF) Beta-Amyloid, Tau, and phosphorylated Tau levels
**Safety**	• Number of adverse events (AEs) according to seriousness, severity, and relationship with EFV.
	• Changes in the liver function panel

8 FDG PET—scan = ^18^Fluorodeoxyglucose Positron emission tomography scan, a-BT = abbreviated Barcelona test, AES = apathy evaluation scale, BDI = Beck’s depression inventory, BNT = Boston naming test, CDR-SoB = clinical dementia rating scale-sum of boxes, C-SSRS = Columbia suicide severity rating scale, DEX = dysexecutive questionnaire, EAT-10 = the eating assessment tool score, EFV = efavirenz, FCSRT = free and cued selective reminding test, JLO = judgement of line orientation test, MRI = magnetic resonance imaging, NPC = Niemann-Pick disease type C, NPI = neuropsychiatric inventory, PDS = Pineda disability scale, SARA = scale for the assessment and rating of ataxia, STROOP = Stroop color and word test, TMT = trail making test, WAIS = Wechsler adult intelligence scale.

A *deterioration in the CDR-SoB* is defined as an increase of ≥ 2 points at 52 weeks of starting treatment with EFV relative to baseline.

A *deterioration in verbal memory* is defined as a decrease of ≥ 1 in the standard deviation (SD) at 52 weeks of starting treatment with EFV relative to baseline.

A *deterioration in the executive function* is defined as a decrease of ≥ 1 in the SD (in at least 2 of the 5 tests performed) at 52 weeks of starting treatment with EFV relative to baseline.

A patient will be considered a responder (no decay in cognitive performance) if there is no deterioration in at least 2 of the 3 assessed items at 52 weeks of starting treatment with EFV relative to baseline.

#### 2.7.1. Study procedure.

Study candidates will be screened during visit 1. Those who meet all the inclusion criteria and none of the exclusion criteria will be invited to participate. Both patient and caregiver must sign the written informed consent, a sine qua non condition for enrollment. Seven study visits will be performed for enrolled patients, and all the information detailed in Table 3 will be gathered.

**Table 3:** Participant timeline.

	Visit 1 (baseline visit)	Visit 2	Visit 2.1	Visit 3	Visit 4	Visit 5	Visit 6
	Screening and enrollment	Start of treatment					End of treatment
	−60/−30 d	Day 0 (Wk 0)	Wk 4 ± 3 d after visit 2	Wk 13 ± 1 wk after visit 2	Wk 26 ± 1 wk after visit 2	Wk 39 ± 1 wk after visit 2	Wk 52 ± 2 wks after visit 2
**Inclusion/exclusion criteria**	✓	✓					
**Written informed consent**	✓						
Demographic data*	✓						
**Vital signs (BP, HR, T, RR) and body weight**	✓	✓	✓	✓	✓	✓	✓
**Physical examination**	✓	✓	✓	✓	✓	✓	✓
**Neurological examination** ‡	✓	✓	✓	✓	✓	✓	✓
**Neuropsychological assessment** §	✓				✓		✓
**General blood tests** ‖	✓			✓	✓	✓	✓
**Biomarkers:** **24-OH-cholesterol**	✓			✓	✓	✓	✓
**Biomarkers:** **Oxysterols** **Lyso-sm-509**	✓				✓		✓
**EKG**	✓			✓	✓	✓	✓
**Medication dispensing**		✓		✓	✓	✓	✓
**Brain MRI**	✓						✓
**Brain ^18^FDG-PET scan**	✓				✓		✓
**Oculography**	✓				✓		✓
**Abdominal ultrasound**	✓				✓		✓
**Lumbar puncture**		✓†					✓†
**AE recording**	✓	✓	✓	✓	✓	✓	✓
**Concomitant medication recording**	✓	✓	✓	✓	✓	✓	✓
**Patient’s journal**		✓		✓	✓	✓	✓
**Pregnancy test**	✓	✓		✓	✓	✓	✓

AE = adverse event, BP = blood pressure, EKG = electrocardiogram, HR = heart rate, MRI = magnetic resonance imaging, RR = respiratory rate, T = temperature.

(1) Includes genetic diagnosis.

‡ Includes Ataxia Scale (SARA), Dysphagia Scale (EAT-10), Pineda Disability Scale.

§ CDR-SoB: Clinical Dementia Rating-Sum of Boxes Scale; a-BT: Abbreviated Barcelona Test; WAIS-III/WAIS-IV: Weschler Adult Intelligence Scale, Cubes, Symbol Search, and Vocabulary subtests; FCSRT: Free and Cued Selective Reminding Test; Verbal Fluency Score: Semantic and phonemic tasks (category fluency [CF] and letter fluency [LF]); TMT: Trail Making Test A and B; SCWD: Stroop Color and Word Test; BNT: Boston Test, subtests Naming and Semantic Knowledge Test; JLO: Judgement of Line Orientation Test; DEX: Dysexecution Questionnaire; NPI: Neuropsychiatric Inventory; AES: Apathy Evaluation Scale; BDI: Beck Depression Scale; C-SSRS: Columbia Suicide Severity Risk Scale.

‖ Complete blood count, coagulation tests, and Biochemistry (sodium, potassium, calcium, protein, glucose, urea, creatinine, aspartate aminotransferase, alanine aminotransferase, cholesterol, TSH, T4).

† **Lumbar puncture** will be performed to determine CSF biomarkers (i.e., Beta-amyloid, Tau, and phosphorylated Tau) in those patients who agree to undergo this procedure. The investigator must inform patients and caregivers that this procedure is optional and about the risks involved. Patients who accept it will be required to sign a separate and specific written informed consent (both patients and caregivers must sign it).

We estimate it will take approximately 3 months to complete the enrollment procedure. We expect to carry out the study between 2022 and 2024.

By the moment of submitting this manuscript, participant recruitment (patients’ screening visit) was still in progress.

Participant Timeline

The EFV will be provided by the study sponsor and will be acquired through the Pharmacy Department at BUH. The supplied EFV is to be used exclusively in this clinical trial. The Pharmacy Department will have an updated record of the EFV and manage an inventory that will collect all product entries, dispensing, returns, and disposals.

The records will comply with current regulations and guidelines. Once they are completed, they must be signed and dated by the person in charge. The clinical trial pharmacist will be responsible for maintaining all records of the medication used. The EFV does not require any special storage conditions according to the SmPC and should not be used after the expiry date indicated on the label.

It is the investigator’s responsibility to ensure that the necessary measures have been taken for proper EFV disposal, as required by applicable legislation, guidelines, and institutional procedures.

### 2.8. Sample size

We have not performed a formal sample size calculation. Adult NPC is an orphan disease, with an estimated prevalence of 27 patients in Spain in 2019. The principal investigator (PI) currently follows 14 of these patients at the BUH. Therefore, we expect to enroll a total of 14 patients.

### 2.9. Recruitment

The study patients will be recruited from the BUH Neurology Department. The PI will recruit patients chronologically, as they come to the BUH, when they meet the selection criteria specified before, until reaching the 14 expected patients.

### 2.10. Assignment of interventions

#### 2.10.1. Allocation: Sequence generation, concealment mechanism, implementation, and blinding (Masking).

Not applicable. This is a single-arm clinical trial; thus, all enrolled patients will receive the study treatment.

### 2.11. Data collection plan

Data collection will be performed during the study visits from both patient interviews and electronic medical registers and will be introduced in an e-CRF created *ad hoc*. A data curation process will be performed to improve data quality and reduce possible errors.

### 2.12. Plans to promote participant retention and complete follow-up

BUH is the reference center of the Spanish National Health System for NPC patients at a national level, and the PI (JG) has been the person in charge of managing these patients for the last years. Therefore, the doctor-patient relationship is very close. Also, to date, only 27 patients have been diagnosed with NPC in Spain. Altogether, this makes it easier to retain the participants and complete the follow-up procedure.

### 2.13. Data management

An electronic case report form (e-CRF) will be created *ad-hoc* for this study based on Research Electronic Data Capture Consortium platform. It will not collect data that allows patient identification.

Comprehensive data will be collected, including the date of patient enrollment, the date of the first administration of EFV, demographic data, medical history, relevant comorbidities, clinical data, any concomitant medications administered, and the results of the biochemical analyses mentioned before.

The study monitoring will be carried out by a Contract Research Organization (OPTIMAPHARM, https://optimapharm.eu/; accessed on August 1st, 2022).

### 2.14. Statistical methods for analyzing primary and secondary outcomes

We will perform a descriptive analysis of the enrolled patients’ baseline sociodemographic and clinical data as a whole and by study groups.

The primary outcome measure will be evaluated by an analysis of covariance (ANCOVA). Cognitive performance (assessed by the previously described neuropsychological test battery at 52 weeks) will be the dependent variable, and the independent variables will be the study group and the cognitive performance assessed by the CDR-SoB at the baseline visit. Tables will be presented with model coefficients and their 95% confidence intervals (95% CI). Additionally, the effect size will be measured by presenting the marginal means per study group and their standardized difference along with their 95% CI.

Given the exploratory nature of the clinical and biological secondary outcome measures, we will use the same statistical technique outlined for the primary outcome measure. However, either generalized models or non-parametric statistics will be used depending on the distribution of the dependent variable.

R version 3.6 or higher will be used to perform the statistical analysis.

### 2.15. Methods for any additional analyses

A descriptive analysis of the AEs recorded throughout the trial will be carried out for patients enrolled in the safety analysis set.

### 2.16. Analysis population and missing data definition of analysis population relating to protocol nonadherence and any statistical methods to handle missing data

In case of missing data, imputation will be made considering the treatment effects’ estimator is not biased and that an increase in type I error has been avoided. However, assuming that the cognitive impairment will slow down and aiming to ensure the trial’s internal sensitivity (in case of expecting a different evolution of one of the experimental groups), the last observation carried forward technique seems to be a conservative approach to the matter.

### 2.17. Oversight and monitoring

#### 2.17.1. Composition of the data monitoring committee, its role and reporting structure.

A Data Safety Monitoring Committee will be established for this trial and will consist of members of the study team and third-party pharmacovigilance experts (from the BUH). The data safety monitoring committee will evaluate all the available EFV safety data at each study visit performed during the trial.

During the development of the study, the sponsor will prepare Drug Safety Update Reports (DSURs) annually, following the recommendations of the ICH E2F guideline, and present them to the regulatory authorities and the IRB following the calendar established in the current legislation.

The sponsor will inform the investigators of any new safety information that could jeopardize the patients’ safety as soon as possible. The investigators will also be notified of any modification to the study protocol.

### 2.18. Description of any interim analyses and stopping guideline

No interim analyses have been planned for this trial.

### 2.19. Adverse event reporting and harms

Given that EFV is an authorized medicinal product, the SmPC will be used as the Reference Safety Information.

The investigators will systematically monitor and collect the AEs from the moment of signing the written informed consent to the patient’s last follow-up visit. AEs will be recorded in the patient’s medical record, stating the causality assessment for EFV. AEs related to the EFV will be collected through the eCRF whenever they are serious (SAEs) or AEs of special interest.

The investigators must provide a thorough description of the event, including start and end dates, an intensity assessment (mild, moderate, or severe), measures taken to mitigate it (e.g., none, EFV withdrawal, the medication used to treat the AE), and a causality assessment for EFV.

Any AE or clinically relevant lab abnormality will be followed until satisfactory resolution, stabilization, or until clinical judgment indicates it has an alternative cause and no further assessment is needed.

The investigator must notify the contracted contract research organization of any SAE (including death from any cause) in less than 24 hour after becoming aware of it, regardless of its relationship with EFV. This will be done by sending a specific notification form by fax or email.

Any suspected unexpected serious adverse reaction (i.e., those not reported in the Reference Safety Information) will be of expedited reporting as per the current Spanish (Royal Decree 1090/2015) and European legislation (Regulation EU 536/2014).

For the purpose of this trial, we consider as AEs of special interests laboratory abnormalities in plasma liver enzymes (aspartate aminotransferase and alanine aminotransferase > 2× upper limits of normal).

In the event of a pregnancy during the study, the EFV will be immediately withdrawn. The investigator must notify the sponsor within 24 hours of becoming aware of the event. Appropriate medical assistance will be offered to the patient. These patients will be asked to sign a specific written informed consent allowing the investigators to collect pregnancy-related data. The patient will be followed until the delivery date; if the outcome meets SAE criteria, the investigators will follow the procedures explained before for SAE notification.

### 2.20. Frequency and procedures for auditing trial conduct

The Investigator shall allow direct access to trial data and documents for monitoring, audits and/or inspections by competent regulatory or health authorities. As such, e-CRFs, source records and other trial-related documentation must be kept current, complete, and accurate at all times.

### 2.21. Ethics approval and consent to participate

The study protocol version 3.0 (February 10th, 2020), EudraCT number 2019-004498-18, received local IRB approval (Ethics and Clinical Investigation Committee of the Bellvitge University Hospital, code AC049/19) on February 27th, 2020). Notably, due to the COVID-19 pandemic and the difficulty obtaining the study medication (EFV), the start of this clinical trial was halted for 2 years. After this period, the protocol was updated to version 3.1 (March 24th, 2020). The local IRB approved this new version on April 15th, 2022. The list of local IRB members is available at https://bellvitgehospital.cat/es/investiga-con-nosotros/ceic/composicion. Accessed on August 1st, 2022.

This Trial will be conducted according to the criteria set by the Declaration of Helsinki (revised on WMA 64th General Assembly, Fortaleza, Brazil, October 2013), good clinical practice standards and applicable regulations. A specific insurance will be hired for this clinical trial.

The level of confidentiality protection, in terms of personal data protection, as required by Spanish (Organic Law on Data Protection 3/2018) and European Law (Regulation EU 2016/679 of the European Parliament and Council), was also ensured (see item 2.25).

Every patient that accepts to participate in the study will be assigned consecutive numbers as they are enrolled, and these numbers (or codes) will be used in the eCRF, instead of personal data. The data collected will be encoded, so that the patient to whom they correspond is not identified.

### 2.22. Plans for communicating important protocol amendments to relevant parties

Major protocol changes will be submitted for IRB approval and minor outcomes will be informed to the IRB. As per good clinical practice, trial participants will be informed of any significant changes during the trial.

### 2.23. Who will obtain informed consent?

The PI (or another physician from the study team) will inform the screened patients about the study and ask them to sign the written informed consent in visit 1 if the patient is interested in participating in the study.

### 2.24. Additional consent provisions for collection and use of participant data and biological specimens

As mentioned before, an optional lumbar puncture will be performed to determine CSF biomarkers (i.e., Beta-amyloid, Tau, and phosphorylated Tau) in those patients who agree to undergo this procedure. An additional written informed consent is required prior to performing the lumbar puncture. Samples will be stored and patients will be informed about the storage along with all study procedures by the recruiting investigator (see item 3.3).

### 2.25. Confidentiality

The results from this clinical trial are confidential and may not be transferred to third parties in any form or manner without written permission from the Sponsor. All individuals involved in the clinical trial are bound to this confidentiality clause in line with the Regulation (EU) 2016/679 of the European Parliament and of the Council of April 27th, 2016, on the protection of natural persons concerning the processing of personal data and the free movement of such data, as well as all other valid and applicable laws and regulations, such as the “Organic Law 3/2018 on Personal Data Protection and Digital Rights Assurance. Therefore, patient data will be anonymized.

While obtaining a signature for the Written Informed Consent, the investigator will request written permission from the patient to directly access his/her data. With this permission granted, the patient’s data may be examined, analyzed, verified, and reproduced for the clinical trial evaluation.

Data will be anonymized so that the corresponding patient cannot be identified. Patient data will also be dissociated. Patients will be assigned consecutive numbers as they are enrolled in the study, and these identification numbers (or codes) will be used in the e-CRF; the patient’s full name will not be included in the e-CRFs. The principal investigator of each center will keep an updated patient identification list containing the name, clinical history number, and the patient’s identification number (or code) for the clinical trial.

The study monitor may have access to the patient’s identity and data related to the study monitoring procedures. Any person with direct access to the data (Regulatory Authorities, Trial Monitors, and auditors) will take all possible precautions to maintain the confidentiality of patients’ identities.

It is the investigator’s responsibility to obtain written informed consent from the study patients. It is the Trial Monitor’s responsibility to make sure that each patient has given his/her written consent to allow this direct access.

The investigator shall ensure that the documents provided to the Sponsor do not contain the patient’s name or any identifiable data.

Dr Cristian Tebé, Head of the Biostatistics Unit, will oversee the final trial dataset, along with the PI.

### 2.26. Ancillary and post-trial care

As mentioned before, BUH is the reference center of the National Health System for NPC patients at a national level, and the PI (JG) has been and still is the person in charge of managing these patients during the last years. Therefore, the PI will remain to be these patients’ assistant physician after the trial ends. On the other hand, a specific insurance has been hired *ad hoc* in case of any harm related to a patient’s participation.

### 2.27. Dissemination policy: trial results, authorship

The study findings will be submitted to a peer-reviewed journal for publication and presented at relevant national and international scientific meetings.

The authorship is based on the criteria established by the International Committee of Medical Journal Editors (https://www.icmje.org/recommendations/browse/roles-andresponsibilities/defining-the-role-of-authors-and-contributors.html, accessed on August 1st, 2022).

### 2.28. Plans to give access to the full protocol, participant level-data and statistical code

The protocol is available on EudraCT Number: 2019-004498-18. No public access to the patient dataset is planned to be given at this moment. Dr Cristian Tebé, the Head of the Biostatistics Unit, will oversee the dataset and granting access to this information will be evaluated on a case-by-case basis and upon reasonable request by the interested part.

## 3. Discussion

NPC is a complex orphan disease, consisting of an unmet medical need for patients suffering from it. Although there are over 20 ongoing studies on NPC registered in the ClinicalTrials.gov database, conducting clinical trials for assessing new therapeutic options can be quite challenging, given the disease’s low prevalence. Different therapeutic approaches are under study (e.g., Miglustat, Arimoclomol, N-Acetyl-L-Leucine), but, to the best of our knowledge, this is the first clinical trial studying the effects of EFV in adult- and late-juvenile-onset NPC.

As mentioned before, neuronal cholesterol metabolism is key for LTP, which is a major event for synaptic plasticity. The enzyme CYP46A1 hydroxylates the cholesterol and mediates the main pathway for its degradation in the brain.^[9,10]^ Given that NPC1 binds cholesterol and participates in its intracellular mobilization, we hypothesized that NPC1 would have a synapse-specific role in redistributing cholesterol and mobilizing CYP46A1 to the synaptic membrane required for LTP. We believe that the capacity of EFV to activate the CYP46A1 could compensate for NPC1 deficiency and correct synaptic changes, therefore compensating cognitive and psychiatric alterations in patients with NPC. This hypothesis is supported by preclinical data^[12,14]^ suggesting that low-dose EFV may be a promising alternative for NPC patients.

On the other hand, this study has limitations. First, the already mentioned small sample size due to the disease’s low prevalence. Therefore, interpreting the results may be tough, given that we cannot safely exclude that a given outcome happened by chance. Second, it has a single-arm design (absence of a control group). Although justifiable by the natural history of the disease, the unexpectedness of spontaneous improvement, and the lack of expected placebo effects, this type of design has idiosyncratic limitations since it could be argued that responders would have responded equally if treated only with SoC. Furthermore, the absence of a frame of reference for comparison could also make the results hard to interpret.

This study may provide direct benefit to enrolled patients in terms of slowing down the disease progression. If the preclinical results are reproducible in a clinical setting, NPC patients may have a new therapeutic option in the future to address their currently unmet medical needs. Nonetheless, future Phase III clinical trials would be needed to confirm the findings of this trial.

*Note: This protocol was written following the SPIRIT statement.^[15]^

### 3.1. Trial status

The current protocol version is 3.1 (March 24th, 2022). This trial is currently on the screening phase (August 2022).

### 3.2. Informed consent materials

Please refer to *Supplementary Material*, http://links.lww.com/MD/I123.

### 3.3. Plans for collection, laboratory evaluation, and storage of biological specimens for genetic or molecular analysis in the current trial and for future use in ancillary studies, if applicable

Biological samples from blood tests and CSF analysis will be analyzed during the conduct of the trial and will be stored in the HUB-ICO-IDIBELL Biobank, which manages and regulates the different biological samples collected at the BUH, Catalan Institute of Oncology (ICO), and other associated centers, under the current Spanish law 14/2007 and the Royal Decree 1716/2011. Additional information on the HUB-ICO-IDIBELL Biobank can be found at https://idibell.cat/en/services/scientific-and-technical-services/biobank/.

Patients will be informed about the biological samples’ storage along with all study procedures by the recruiting investigator. This information will also be displayed in the Patient Information Sheet, and all patients must sign the written informed consent prior to any study-related procedure, including the storage of any biological sample.

## Acknowledgments

We thank the *Spanish Niemann-Pick Foundation* for providing financial support. We also thank the *Bellvitge University Hospital, IDIBELL and CERCA Program/Generalitat de Catalunya* for institutional support.

## Author contributions

**Conceptualization:** Jordi Gascón-Bayarri, Petru Cristian Simon, Sebastián Videla.

**Funding acquisition:** Jordi Gascón-Bayarri, María Dolores Ledesma, Sebastián Videla.

**Investigation:** Jordi Gascón-Bayarri, Imma Rico, Cristina Sánchez-Castañeda, Jaume Campdelacreu-Fumadó, Nahum Calvo-Malvar, Mònica Cos, Eugenia de Lama, Montserrat Cortés-Romera, Laura Rodríguez-Bel, Celia Pérez-Sousa, María Cerdán Sánchez, Nuria Muelas, María Dolores Sevillano, Pablo Mir, Adolfo López de Munain, Anna Ferrer.

**Methodology:** Jordi Gascón-Bayarri, Petru Cristian Simon, Thiago Carnaval, Sebastián Videla.

**Supervision:** Jordi Gascón-Bayarri, Roser Llop.

**Writing – original draft:** Jordi Gascón-Bayarri, Thiago Carnaval, Sebastián Videla.

**Writing – review & editing:** Jordi Gascón-Bayarri, Petru Cristian Simon, Thiago Carnaval, María Dolores Ledesma, Sebastián Videla.

## Supplementary Material


