# Supplementary material for: Efficacy and safety clinical trial with efavirenz in patients diagnosed with adult Niemann-pick type C with cognitive impairment
Source: Medicine (Baltimore). 2022 Dec 2;101(48):e31471. doi: 10.1097/MD.0000000000031471 (PMC9726274; doi:10.1097/MD.0000000000031471)
Supplement: Supplementary file 1 [file medi-101-e31471-s001.pdf]

Hoja de información al paciente y Documento de consentimiento informado

**Título del estudio:**	"Ensayo clínico de eficacia y seguridad de efavirenz en pacientes diagnosticados de Niemann-Pick C del adulto con deterioro cognitivo"
**Número del protocolo:**	HUB-NEU-2019-01
**Promotores:**	Dr. Jordi Gascón<br>Dra. Lola Ledesma
**Investigador principal:**	Dr. Jordi Gascón.<br>Servicio de Neurología. Hospital Universitari de Bellvitge<br>Tel.: 93 2607628
**Centro:**	Hospital Universitari de Bellvitge<br>Carrer de la Feixa Llarga, s/n<br>08907-L'Hospitalet de Llobregat<br>Barcelona

Introducción

Nos dirigimos a usted para informarle sobre un estudio de investigación en el que se le invita a participar. El estudio ha sido aprobado por un Comité de Ética de la Investigación con medicamentos y por la Agencia Española de Medicamentos y Productos Sanitarios, de acuerdo a la legislación vigente, el Real Decreto 1090/2015 de 4 de diciembre y el Reglamento Europeo 536/2014 de 16 de abril, por los que se regulan los ensayos clínicos con medicamentos. El ensayo clínico se llevará a cabo acorde con las Normas de Buena Práctica Clínica y se respetará la Declaración de Helsinki.

Nuestra intención es que usted reciba la información correcta y suficiente para que pueda decidir si acepta o no participar en este estudio. Para ello lea esta hoja informativa con atención y nosotros le aclararemos las dudas que le puedan surgir.

Además, puede tomarse el tiempo necesario para consultarlo con otros familiares o personas relacionadas antes de otorgar el consentimiento informado.

Participación voluntaria

Le invitamos a participar en el estudio porque ha sido diagnosticado de la enfermedad de Niemann Pick tipo C (NPC) del adulto.

Debería saber que su participación en este estudio es voluntaria y que puede decidir NO participar. Si decide participar, puede cambiar su decisión y retirar el consentimiento en cualquier momento, sin que por ello se altere la relación con su médico ni se produzca perjuicio alguno en su atención sanitaria. En caso de retirarse del estudio, puede hacerlo sin dar ningún tipo de explicación, ni pérdida de ningún derecho.

Objetivo del estudio

El objetivo del estudio es evaluar la eficacia y seguridad de Efavirenz a dosis bajas (25 mg al día), por vía oral, añadido al tratamiento habitual, en pacientes diagnosticados de NPC del adulto con deterioro cognitivo.

Descripción del estudio

En el presente estudio se incluirán a pacientes de ambos sexos, con edad mayor o igual a 14 años, diagnosticados de NPC con deterioro cognitivo ligero-moderado, que reciben tratamiento habitual con Miglustat a una dosis estable durante los últimos 3 meses.

La enfermedad de NPC del adulto con deterioro cognitivo es una enfermedad genética neurodegenerativa, sin tratamiento curativo y con una sola opción terapéutica con una eficacia limitada (Miglustat, *Zavesca*®). En el año 2019, la prevalencia de NPC del adulto en España es de 27 pacientes. En el presente estudio participará un solo centro, el Hospital Universitari de Bellvitge, por lo que se prevé incluir a todos los pacientes visitados en el Servicio de Neurología del centro (un total de aproximadamente 14 pacientes).

El fármaco que se propone estudiar es **Efavirenz**, un medicamento autorizado y comercializado en España para el tratamiento del virus de la inmunodeficiencia humana-1 (VIH-1) en adultos infectados, adolescentes y niños ≥3 meses de edad. En adultos, la dosis recomendada de Efavirenz (en combinación con otros medicamentos) es de 600 mg, una vez al día, por vía oral. En el presente estudio se evaluará una dosis 24 veces inferior (de 25 mg al día), pero que ha resultado eficaz y segura en estudios

preclínicos en animales. Se trata de un estudio de un solo grupo de tratamiento, en el cual todos los pacientes recibirán el mismo tratamiento: Efavirenz 25 mg al día, por vía oral, aparte de la medicación habitual con Miglustat.

Actividades del estudio

La duración del estudio es de 52 semanas (1 año). Durante el estudio, debería acudir a 6 visitas:

- Visita 1 (de cribaje e inclusión del paciente)
- Visita 2 (entre los 30 y 60 días de la visita 1, corresponde al inicio del tratamiento, día 0)
- Visita 3 (semana 13 ±1 semana de la visita 2)
- Visita 4 (semana 26 ±1 semana de la visita 2)
- Visita 5 (semana 39±1 semana de la visita 2)
- Visita 6 (visita fin de estudio, semana 52±2 semanas de la visita 2)

Durante las visitas:

- se medirán las constantes vitales (tensión arterial, frecuencia cardíaca, frecuencia respiratoria, etc);

- se realizarán exploraciones físicas, neurológicas y neuropsicológicas. Las exploraciones neurológicas y neuropsicológicas consisten en responder a varios cuestionarios relacionados con la enfermedad;

- se recogerán muestras de sangre en la primera, tercera, cuarta, quinta y sexta visita, lo que corresponde con la práctica clínica habitual;

- se efectuará electrocardiograma, lo que corresponde con la práctica clínica habitual en algunos pacientes;

- se realizarán resonancias magnéticas craneales en la primera y última visita, lo que corresponde con la práctica clínica habitual;

- se realizarán tomografías craneales (tomografías con emisión de positrones o PET) en la primera, cuarta y última visita del estudio. La primera y la última corresponden con la práctica clínica habitual, la única PET *extra* (es decir, que no se realiza en la práctica clínica) será la de la cuarta visita, que se realizará a raíz de la participación en el estudio;

- se efectuarán oculografías en la primera, cuarta y última visita, que corresponden con la práctica clínica habitual;

- se realizarán ecografías del abdomen en la primera, cuarta y última visita, que corresponden con la práctica clínica habitual;
- se efectuará una punción lumbar en la segunda y última visita. Solamente una punción lumbar corresponde con la práctica clínica habitual, la otra será *extra*, a raíz de la participación en el estudio;
- y se recogerán los datos sobre efectos adversos a Efavirenz.

En la siguiente tabla se detallan los procedimientos que se realizarán en cada visita:

	**Visita 1**	**Visita 2**	**Visita 3**	**Visita 4**	**Visita 5**	**Visita 6**
	Inclusión paciente	Inicio tratamiento				Fin de estudio
	-60/30 Días	Día 0	Semana 13	Semana 26	Semana 39	Semana 52
Constantes vitales	✓	✓	✓	✓	✓	✓
Explor. físicas	✓	✓	✓	✓	✓	✓
Explor. neurológicas	✓	✓	✓	✓	✓	✓
Explor. neuropsicológ.	✓			✓		✓
Analíticas sangre	✓		✓	✓	✓	✓
Electrocardiograma	✓		✓	✓	✓	✓
Resonancia craneal	✓					✓
PET craneal	✓			✓		✓
Oculografia	✓			✓		✓
Ecografía abdominal	✓			✓		✓
Punción lumbar		✓				✓
Registro efectos adv.	✓	✓	✓	✓	✓	✓
Test embarazo		✓				✓

En resumen, todas las pruebas que se le van a realizar en este estudio corresponden a las que se le realizaría dentro de la práctica clínica, excepto las dos siguientes: una PET craneal a la mitad del estudio y una punción lumbar al final del estudio.

Riesgos y molestias derivados de su participación en el estudio

Efavirenz está autorizado y comercializado en España para el tratamiento de pacientes adultos, adolescentes y niños ≥3 meses de edad, infectados por VIH. Según la ficha técnica, en adultos con VIH, la dosis recomendada de Efavirenz es de 600 mg al día.

Respecto a su perfil de seguridad, Efavirenz se ha estudiado en más de 9.000 pacientes. Las reacciones adversas notificadas más frecuentemente, consideradas como mínimo de gravedad moderada, notificadas en al menos un 5% de los pacientes que recibieron dosis de 600 mg al día de Efavirenz fueron: erupción cutánea, mareos, náuseas, cefalea y fatiga. Las reacciones adversas más relevantes asociadas a Efavirenz son las reacciones cutáneas y los síntomas del sistema nervioso. Los síntomas del sistema nervioso generalmente empiezan inmediatamente después del inicio del tratamiento y generalmente se resuelven después de las primeras 2-4 semanas. Entre las reacciones cutáneas graves se incluyen el síndrome de Stevens-Johnson y el eritema multiforme. Aparte, se han visto reacciones adversas psiquiátricas incluyendo depresión, psicosis, manía, ideas e intentos de autolisis.

Al ser un fármaco aprobado por las autoridades sanitarias competentes, existe información al acceso de todo el mundo sobre los efectos secundarios de Efavirenz. Por favor, hable con el médico de su estudio para obtener una lista completa de los efectos secundarios comunicados con este fármaco y en cualquier caso se le podrá facilitar el prospecto del fármaco.

La dosis que se utilizará en el presente estudio es 24 veces menor (de 25 mg al día) que la de la ficha técnica. La dosis de 100 mg/d corresponde a la dosis utilizada en la población pediátrica de 3,5 a 5 kg de peso. Durante el estudio, y según los valores del laboratorio clínico, es posible aumentar la dosis de Efavirenz como máximo hasta 200 mg al día. Aún así, no se espera que haya problemas de seguridad con estos niveles de dosis.

Entre las “pautas a seguir” que debería cumplir si decide participar en el estudio serían el cumplimiento con las visitas / actividades del estudio y la respuesta a varios cuestionarios relacionados con la enfermedad. Los riesgos derivados a las pruebas realizadas durante las visitas del estudio puede usted consultarlas en el apartado procedimiento de obtención de muestras, molestias y posibles riesgos.

Debería avisar al médico responsable del estudio de cualquier evento adverso que le suceda. No modifique la medicación que está tomando ni tome otros medicamentos o “plantas medicinales” sin consultar antes con el médico del estudio.

Posibles beneficios

Se espera que el tratamiento con Efavirenz aporte una mejoría en el rendimiento cognitivo

al año de tratamiento. Pero, también, es posible que no obtenga ningún beneficio para su salud por participar en este estudio.

Advertencia relativa al embarazo

Efavirenz no se debe utilizar durante el embarazo. A las mujeres en edad fértil que participen en el estudio se les realizará a una prueba de embarazo antes de iniciar el tratamiento con Efavirenz y se les recomendará utilizar siempre anticonceptivos de barrera en combinación con otros métodos anticonceptivos (p. ej., anticonceptivos orales u otros anticonceptivos hormonales) o la abstención sexual al menos hasta 12 semanas después de la última dosis de fármaco. En el caso de los varones incluidos en el estudio, debería asegurarse el uso de anticonceptivos de barrera al menos hasta 12 semanas después de la última dosis de fármaco.

En caso de producirse un embarazo durante su participación en el estudio, debería informar a su médico de inmediato para recibir la asistencia médica adecuada. En caso de producirse un embarazo, se solicitará la recogida de datos del mismo y de datos de salud del bebé hasta 12 semanas después del parto. Se garantizará el cumplimiento de la Ley Orgánica 3/2018, de 5 de diciembre, sobre protección de datos personales y garantía de los derechos digitales.

Tratamientos alternativos

La enfermedad de NPC del adulto con deterioro cognitivo es una enfermedad sin tratamiento curativo y con una sola opción terapéutica con una eficacia limitada (Miglustat, Zavesca®). Usted, aparte de Efavirenz, seguirá recibiendo el tratamiento con Miglustat durante el presente estudio. En principio, no se le retirará el tratamiento habitual con Miglustat.

Póliza de seguro

El Promotor del estudio dispone de una póliza de seguros que se ajusta a la legislación vigente (Real Decreto 1090/2015) y que le proporcionará la compensación e indemnización en caso de menoscabo de su salud o de lesiones que pudieran producirse en relación con su participación en el estudio, siempre que no sean consecuencia de la propia enfermedad que se estudia o de la evolución propia de su enfermedad, como consecuencia de la ineficacia del tratamiento.

Si desea más información relativa a este apartado, consulte con el investigador principal del estudio en su centro.

Le informamos que es posible que su participación en este ensayo clínico pueda modificar las condiciones generales y particulares (cobertura) de sus pólizas de seguros (vida, salud, accidente). Por ello, le recomendamos que se ponga en contacto con su aseguradora para determinar si la participación en este estudio afectará a su actual póliza de seguros.

Protección de datos personales

El promotor se compromete al cumplimiento del Reglamento UE 2016/679 del Parlamento Europeo y del Consejo de 27 de abril de 2016 de Protección de Datos (RGPD). También, el promotor se compromete al cumplimiento de la Ley Orgánica 3/2018, de 5 de diciembre, sobre protección de datos personales y garantía de los derechos digitales. Los datos recogidos para el estudio estarán identificados mediante un código, de manera que no incluya información que pueda identificarle, y sólo su médico del estudio/colaboradores podrá relacionar dichos datos con usted y con su historia clínica. Por lo tanto, su identidad no será revelada a persona alguna salvo excepciones en caso de urgencia médica o requerimiento legal. El tratamiento, la comunicación y la cesión de los datos de carácter personal de todos los participantes se ajustarán a lo dispuesto en esta ley.

El acceso a su información personal identificada quedará restringido al médico del estudio/colaboradores, autoridades sanitarias (Agencia Española de Medicamentos y Productos Sanitarios, autoridades sanitarias extranjeras), al Comité de Ética de la Investigación y personal autorizado por el promotor (monitores del estudio, auditores), cuando lo precisen para comprobar los datos y procedimientos del estudio, pero siempre manteniendo la confidencialidad de los mismos de acuerdo a la legislación vigente.

Los datos se recogerán en un fichero de investigación responsabilidad de la institución y se tratarán en el marco de su participación en este estudio. El promotor adoptará las medidas pertinentes para garantizar la protección de su privacidad y no permitirá que sus datos se crucen con otras bases de datos que pudieran permitir su identificación. De acuerdo a lo que establece la legislación de protección de datos, usted puede ejercer los derechos de *acceso, modificación, oposición y cancelación* de datos, para lo cual debería dirigirse a su médico del estudio. Además también puede limitar el tratamiento de datos que sean incorrectos, solicitar una copia o que se trasladen a un tercero (portabilidad) los datos que usted ha facilitado para el estudio. Para ejercitar sus derechos, diríjase al investigador principal del estudio o al Delegado de protección de datos de la institución, email: dataprotection@idibell.cat.

Si usted decide retirar el consentimiento para participar en este estudio, ningún dato nuevo será añadido a la base de datos, pero sí se utilizarán los que ya se hayan recogido.

Los datos codificados pueden ser transmitidos a terceros y a otros países pero en ningún caso contendrán información que le pueda identificar directamente, como nombre y apellidos, iniciales, dirección, nº de la seguridad social, etc. En el caso de que se produzca esta cesión, será para los mismos fines del estudio descrito o para su uso en publicaciones científicas pero siempre manteniendo la confidencialidad de los mismos de acuerdo a la legislación vigente.

El Investigador y el Promotor están obligados a conservar los datos recogidos para el estudio al menos hasta 25 años tras su finalización. Posteriormente, su información personal solo se conservará por el centro para el cuidado de su salud y por el promotor para otros fines de investigación científica si usted hubiera otorgado su consentimiento para ello y si así lo permite la ley y requisitos éticos aplicables.

Si se realizara una transferencia de sus datos codificados fuera de la UE a las entidades de nuestro grupo, a prestadores de servicios o a investigadores científicos que colaboren con nosotros, los datos del participante quedarán protegidos con salvaguardas tales como contratos u otros mecanismos por las autoridades de protección de datos. Si el participante quiere saber más al respecto, puede contactar al Delegado de Protección de Datos del Promotor (jordigneuro@bellvitgehospital.cat).

Gastos y compensación económica

El promotor del estudio es el responsable de gestionar la financiación del mismo.

Usted no tendrá que pagar por los medicamentos ni por pruebas específicas del estudio. Su participación en el estudio no le supondrá ningún gasto adicional.

Otra información relevante

Una descripción de este ensayo clínico estará disponible en http://reec.aemps.es, según exige la legislación española.

Cualquier nueva información referente a los fármacos utilizados en el estudio y que pueda afectar a su disposición para participar en el estudio, que se descubra durante su participación, le será comunicada por su médico lo antes posible.

Debería saber que puede ser excluido del estudio si el promotor o los investigadores del estudio lo consideran oportuno, ya sea por motivos de seguridad, por cualquier acontecimiento adverso que se produzca por la medicación en estudio o porque consideren que no está cumpliendo con los procedimientos establecidos. En cualquiera de los casos, usted recibirá una explicación adecuada del motivo que ha ocasionado su retirada del estudio.

Al firmar la hoja de consentimiento adjunta, usted se debería comprometer a cumplir con los procedimientos del estudio que se le han expuesto.

Debería usted saber que es posible que su médico de Atención Primaria tenga conocimiento de su participación en este estudio.

¿Qué tratamiento recibiré cuando finalice el ensayo clínico?

Una vez finalizado el ensayo clínico, se les ofrecerá la posibilidad de continuar el tratamiento con Efavirenz en un estudio posterior, de extensión, cuyo protocolo se presentará también al Comité de Ética de la Investigación con medicamentos para su evaluación. El estudio de extensión se iniciará antes de conocer los resultados del presente ensayo clínico, siempre y cuando el equipo investigador no haya observado problemas de seguridad con Efavirenz. Por lo tanto, usted no se verá obligado a interrumpir el tratamiento con Efavirenz una vez acabado el presente ensayo clínico.

Contacto en caso de dudas

Si durante su participación tiene alguna duda o necesita obtener más información, póngase en contacto con el Dr. Jordi Gascón del Servicio de Neurología del Hospital Universitari de Bellvitge (tel.: 93 2607628).

Estudios clínicos en menores de edad

En el caso de que su edad sea menor a 18 años, le informamos que se le va a entregar a su hijo una hoja de información y asentimiento informado adaptados a su capacidad de entendimiento que debería firmar.

El artículo 156 del Código Civil se especifica que «El documento de consentimiento informado de los padres será válido siempre que vaya firmado por uno de ellos con el consentimiento expreso o tácito del otro que debe quedar suficientemente documentado».

☐ Los progenitores (ambos)

En el supuesto de que autorizase solo uno de los progenitores, el progenitor que autoriza habrá de declarar una de las siguientes:

☐ Confirmo con la presente que el otro progenitor no se opone a la participación de nuestro hijo/a en el estudio.

☐ El firmante es el único tutor legal.

Obtención y utilización de muestras biológicas

Objetivos

Su participación en este ensayo clínico conlleva la obtención y utilización de muestras biológicas con fines de investigación, para lo que se observará la Ley 14/2007 de investigación biomédica y el Real Decreto 1716/2011, normativas que garantizan el respeto a los derechos que le asisten. Al firmar este documento, revisado y evaluado favorablemente por el Comité de Ética de la Investigación con medicamentos que ha aprobado este ensayo clínico, usted acepta que se utilicen sus muestras para las finalidades del presente estudio.

Durante el estudio se obtendrán muestras biológicas en sangre y en el líquido cefalorraquídeo (por punción lumbar). **Las punciones lumbares son opcionales.** Se trata de la evaluación de biomarcadores bioquímicos relacionados con su enfermedad: Oxisteroles, Lyso-Sm-509 y 24-OH-Colesterol (en sangre) y Beta-Amiloide, Tau y Tau fosforilada (en líquido cefalorraquídeo).

Procedimientos de obtención de muestras, molestias y posibles riesgos

Algunas de las muestras se obtienen durante el seguimiento habitual de su enfermedad o proceso; otras son solicitadas porque son necesarias para cumplir con los objetivos de este estudio. A continuación le explicamos cuáles son y los riesgos asociados a los procedimientos utilizados para su obtención:

- Muestras de sangre: se obtendrán 5 muestras de sangre y la cantidad extraída en cada análisis será de aprox. 10 ml. de sangre. En general, las punciones con agujas para la extracción de sangre no suponen ningún problema. Sin embargo, en ocasiones, pueden provocar hemorragias, hematomas, molestias, infecciones y/o dolor en el punto de extracción de sangre. También puede sentirse mareado.
- Muestras de líquido cefalorraquídeo: se obtendrán 2 muestras de líquido cefalorraquídeo por punción lumbar**, que son opcionales**, y la cantidad extraída en cada análisis será de aprox. 10 ml. de líquido. En general, las punciones con agujas para la extracción de líquido cefalorraquídeo no suponen ningún problema. Sin embargo, en ocasiones, pueden provocar hemorragias, hematomas, molestias, infecciones y/o dolor en el punto de extracción de líquido cefalorraquídeo. También puede sentirse mareado o tener dolor de cabeza después de la punción.

Los posibles riesgos derivados del procedimiento realizado para la obtención de estas muestras estarán cubiertos por el seguro del ensayo clínico.

Las muestras estarán asociadas a un código que solo podrá ser relacionado con su identidad por personal autorizado, de la misma manera que se ha explicado previamente con los datos obtenidos durante el ensayo. Los datos que se deriven de la utilización de estas muestras se tratarán del mismo modo que el resto de datos que se obtengan durante este ensayo (ver apartado de protección de datos personales).

Las muestras y los datos asociados se mantendrán bajo las condiciones de seguridad adecuadas y se garantiza que los sujetos no podrán ser identificados a través de medios considerados razonables por personas distintas a las autorizadas.

Beneficios esperados

No se espera un beneficio directo por su participación en el estudio. No obstante, los conocimientos obtenidos gracias a los estudios llevados a cabo a partir de sus muestras y de muchas otras pueden ayudar al avance médico y, por ello, a otras personas. No percibirá ningún beneficio económico por la donación de las muestras y la cesión de los datos proporcionados, ni tendrá derechos sobre posibles beneficios comerciales de los descubrimientos que puedan conseguirse como resultado de la investigación efectuada.

Lugar de análisis y almacenamiento de muestras

Durante el desarrollo del ensayo, sus muestras pueden ser analizadas en diversos laboratorios (Hospital Universitario de Bellvitge, Hospital Clinic i Provincial de Barcelona, CBM Severo Ochoa de Madrid y el Laboratorio Centogen de Alemania) y se mantendrán almacenadas hasta la finalización del ensayo, en previsión de que fuera necesario repetir algún análisis adicional relacionado con los objetivos del ensayo. Durante este proceso el responsable de las muestras será el promotor del ensayo.

Uso futuro de las muestras

Una vez finalizado el ensayo, las muestras sobrantes serán destruidas, a no ser que usted consienta para que puedan ser almacenadas y utilizadas en futuras investigaciones.

Derecho de revocación del consentimiento

Si cambiara de opinión en relación con la donación de las muestras biológicas y la cesión de los datos proporcionados, tiene derecho a solicitar su destrucción o anonimización, a través de

su médico. No obstante, debe saber que los datos que se hayan obtenido en los análisis realizados hasta ese momento podrán ser utilizados para los fines solicitados y podrán conservarse en cumplimiento de las obligaciones legales correspondientes.

Implicaciones de la información obtenida al analizar las muestras

En el caso de que usted lo solicite, se le podrá facilitar información acerca de los estudios de investigación en los que se hayan utilizado sus muestras, así como de los resultados generales del presente ensayo.

En el caso de que en este ensayo se obtengan datos que pudieran ser clínicamente relevantes para usted, e interesar a su salud o a la de su familia, podrá solicitar que le sean comunicados por su médico del ensayo si así lo indica en la casilla que aparece al final de este documento. No obstante, si el paciente hubiera indicado su negativa y cuando esta información, según criterio del médico responsable, sea necesaria para evitar un grave perjuicio para su salud o la de sus familiares biológicos, se informará a un familiar próximo o a un representante, previa consulta al Comité de Ética Asistencial del centro. La comunicación de esta información se llevará a cabo por profesionales que le podrán explicar adecuadamente su relevancia y las opciones que se pudieran plantear.

En el caso de donante menor de edad, llegada la mayoría de edad tendrá derecho a recibir esta información y a la revocación del consentimiento. En caso de que no lo ejerza, se considerará que el actual documento sigue vigente.

En relación a las punciones lumbares opcionales. Consiento a la realización de las punciones lumbares específicas explicadas en la hoja de información:

☐ SI ☐ NO

Consiento a ser contactado en el caso de necesitar más información o muestras biológicas adicionales:

☐ SI ☐ NO Teléfono o e-mail de contacto…………………….

Hoja de Consentimiento de Participante

CONSENTIMIENTO INFORMADO

**Título del estudio:**	“Ensayo clínico de eficacia y seguridad de Efavirenz en pacientes diagnosticados de Niemann-Pick C del adulto con deterioro cognitivo”
**Número del protocolo:**	HUB-NEU-2019-01

Yo, *<<nombre y apellidos del participante>>*

☐ He leído la hoja de información que se me ha entregado sobre el estudio.

☐ He podido hacer preguntas sobre el estudio.

☐ He recibido suficiente información sobre el estudio.

☐ He hablado con el/la dr/a. …………………………..

☐ Comprendo que mi participación es voluntaria.

☐ Comprendo que puedo retirarme del estudio:

- Cuando quiera.
- Sin tener que dar explicaciones.
- Sin que esto repercuta en mis cuidados médicos.

Recibiré una copia firmada y fechada de este documento de consentimiento informado.

Presto libremente mi conformidad para participar en el estudio.

Firma del participante

Fecha:_____/____/_____

Firma del investigador

Fecha:_____/____/_____

Firma del representante legal,
familiar o persona vinculada de hecho

Fecha:_____/____/_____

Firma del investigador

Fecha:_____/____/_____

Deseo que me comuniquen la información derivada de la investigación que pueda ser relevante para mí salud:

☐ SÍ Teléfono o e-mail de contacto…………………….

☐ NO

Firma del participante Firma del investigador

Fecha:____/____/____ Fecha:____/____/____

Hoja de Consentimiento de Participante ante testigos

CONSENTIMIENTO INFORMADO

**Título del estudio:**	"Ensayo clínico de eficacia y seguridad de efavirenz en pacientes diagnosticados de Niemann-Pick C del adulto con deterioro cognitivo"
**Número del protocolo:**	HUB-NEU-2019-01

Yo, <<*nombre y apellidos del testigo*>>, como testigo, afirmo que en mi presencia se ha informado a D/Dª <<*nombre y apellidos del participante*>> y se ha leído la hoja de información que se le ha entregado sobre el estudio, de modo que:

☐ Ha podido hacer preguntas sobre el estudio.

☐ Ha recibido suficiente información sobre el estudio.

☐ Ha hablado con el/la dr/a. …………………………..

☐ Comprende que su participación es voluntaria.

☐ Comprende que puede retirarse del estudio:

- Cuando quiera.
- Sin tener que dar explicaciones.
- Sin que esto repercuta en sus cuidados médicos.

Recibiré una copia firmada y fechada de este documento de consentimiento informado.

Firma del testigo   Firma del investigador

Fecha:____/____/____   Fecha:____/____/____

El participante desea que le comuniquen la información derivada de la investigación que pueda ser relevante para su salud:

☐ SÍ Teléfono o e-mail de contacto…………………….

☐ NO

Firma del testigo

Fecha:____/____/____

Firma del investigador

Fecha:____/____/____
